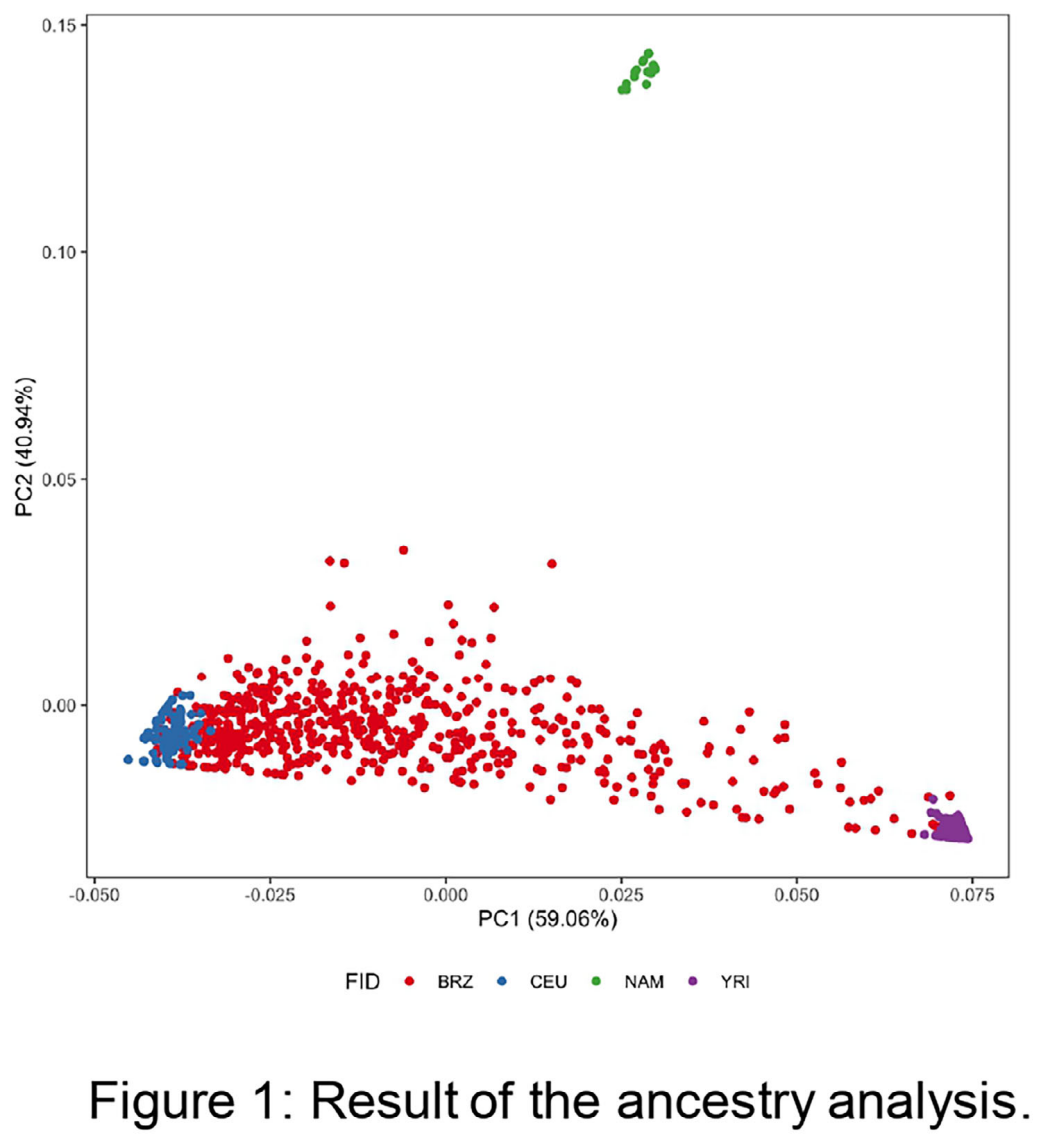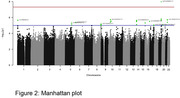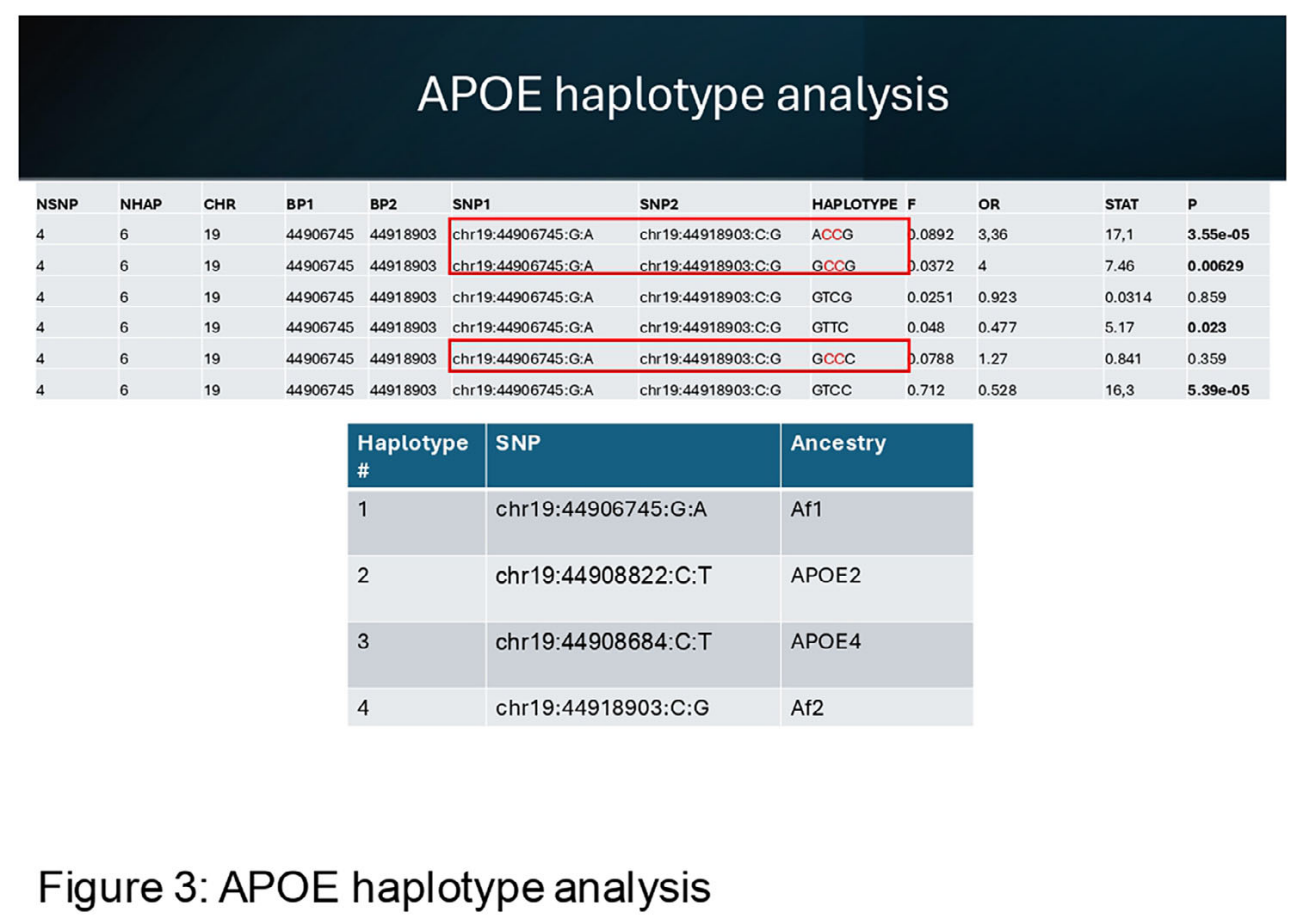# African ancestry component could change APOE4 effect size for AD of a diverse population

**DOI:** 10.1002/alz.091733

**Published:** 2025-01-03

**Authors:** Maria Aparecida Camargos Bicalho, BERNARDO DE MATTOS VIANA, Marco Túlio Cintra, Erika de Oliveira Hansen, Ivonne Carolina Bolaños Burgos, Gabriela Tomé Oliveira Engelmann, Debora Marques de Miranda, Marco Aurélio Romano‐Silva, Kayenat Parveen, Maria Carolina Dalmasso, Luiz Armando Cunha de Marco, Alfredo Ramirez

**Affiliations:** ^1^ Graduate Program in Applied Sciences to Adult Health, Faculdade de Medicina, Universidade Federal de Minas Gerais, Belo Horizonte Brazil; ^2^ Federal University of Minas Gerais, Belo Horizonte, Minas Gerais Brazil; ^3^ National Institute of Science and Technology Neurotec R (INCT‐MM), Faculdade de Medicina, Universidade Federal de Minas Gerais, Belo Horizonte Brazil; ^4^ Department of Clinical Medicine, Faculdade de Medicina, Universidade Federal de Minas Gerais, Belo Horizonte Brazil; ^5^ Older Adult’s Psychiatry and Psychology Extension Program I Federal University of Minas Gerais, Belo Horizonte, Minas Gerais Brazil; ^6^ INCT – NeuroTecR and CTMM, Belo Horizonte, Minas Gerais Brazil; ^7^ UFMG, Belo Horizonte Brazil; ^8^ Graduate Program in Molecular Medicine, Belo Horizonte, Minas Gerais Brazil; ^9^ INCT – NeuroTecR and CTMM, Belo Horizonte, MG Brazil; ^10^ Older Adult’s Psychiatry and Psychology Extension Program I Federal University of Minas Gerais, Belo Horizonte, MG Brazil; ^11^ Universidade Federal de Minas Gerais, Belo Horizonte Brazil; ^12^ National Institute of Science and Technology ‐ NeuroTecR and CTMM, Belo Horizonte, MG Brazil; ^13^ Department of Mental Health, School of Medicine, Federal University of Minas Gerais, Belo Horizonte, MG Brazil; ^14^ Molecular Medicine Program, Faculdade de Medicina, Universidade Federal de Minas Gerais, Belo Horizonte Brazil; ^15^ Jenny de Andrade Faria Institute – Outpatient Reference Center for the Elderly, Universidade Federal de Minas Gerais (UFMG), Belo Horizonte, Minas Gerais Brazil; ^16^ graduate program in applied sciences to adult health, Belo Horizonte, Minas Gerais Brazil; ^17^ National Institute of Science and Technology Neurotec R (INCT‐MM) Faculdade de Medicina, Universidade Federal de Minas Gerais, Belo Horizonte Brazil; ^18^ Molecular Medicine Program, Faculdade de Medicina, Belo Horizonte Brazil; ^19^ Neurotec R National Institute of Science and Technology (INCT‐Neurotec R), Faculty of Medicine, Universidade Federal de Minas Gerais (UFMG), Belo Horizonte, Minas Gerais Brazil; ^20^ Molecular Medicine Postgraduate Program, Faculty of Medicine, Universidade Federal de Minas Gerais (UFMG), Belo Horizonte, Minas Gerais Brazil; ^21^ Department of Neurodegenerative Diseases and Geriatric Psychiatry, University Hospital Bonn, Bonn Germany; ^22^ Division of Neurogenetics and Molecular Psychiatry, Department of Psychiatry and Psychotherapy, Faculty of Medicine and University Hospital Cologne, University of Cologne, Cologne Germany; ^23^ Studies in Neuroscience and Complex Systems ENyS‐CONICET‐HEC‐UNAJ, Florencio Varela Argentina; ^24^ National Institute of Science and Technology Neurotec R (INCT‐MM), Faculdade de Medicina, Belo Horizonte Brazil; ^25^ Department of Surgery, Universidade Federal de Minas Gerais, Belo Horizonte, Minas Gerais Brazil; ^26^ Glenn Biggs Institute for Alzheimer’s and Neurodegenerative Diseases, San Antonio USA; ^27^ Department of Neurodegenerative Diseases and Geriatric Psychiatry, University of Bonn Medical Center, Bonn Germany; ^28^ University of Bonn, Bonn Germany; ^29^ Cluster of Excellence Cellular Stress Responses in Aging‐Associated Diseases (CECAD), University of Cologne, 50931, Cologne Germany; ^30^ Department of Psychiatry & Glenn Biggs Institute for Alzheimer’s and Neurodegenerative Diseases, San Antonio, TX USA; ^31^ Division of Neurogenetics and Molecular Psychiatry, Department of Psychiatry and Psychotherapy, University of Cologne, Medical Faculty, Cologne Germany; ^32^ University of Bonn Medical Center, Dept. of Neurodegenerative Disease and Geriatric Psychiatry/Psychiatry, Bonn Germany; ^33^ German Center for Neurodegenerative Diseases (DZNE), Bonn Germany

## Abstract

**Background:**

Worldwide, the actual number of 55 million people diagnosed with dementia is estimated to increase to 139 million people affected by dementia in 2050. 61% of these individuals resided in low and middle‐income countries (LMIC). Genetic risk factors account for up to 80% of the attributable risk of Alzheimer’s disease (AD), the leading cause of dementia. Thus, one can argue that most of the pathophysiological pathways in AD are driven by genetic determinants. Despite the increasing number of dementia cases in LMIC, poor generalizability of genetic studies across populations arises from the abundance of studies focused on European descent and the lack of studies in globally diverse populations.

**Method:**

We developed a case control study, including older adults selected from Cog‐aging Research Group of the UFMG, Brazil, divided in two groups: control and AD (AD and AD plus vascular dementia). They were submitted to the same neuropsychological and geriatric protocols. Diagnosis of AD dementia were based on Mckhann criteria. DNA samples were subjected to genome‐wide‐genotyping using the Infinium Global Screening Array (Illumina). Quality controls were performed using PLINK and R. After QC, remaining samples were analyzed for population stratification. TOPMed imputation server was used to perform imputation. Principal components (PC) analysis considered phenotype, sex, and age.

**Result:**

The final sample was composed by 573 samples, 181 controls and 309 cases, 389 female and 184 males. Our sample was composed predominantly by Caucasian and African genetic ancestries (Figure 1). From 23 top hit variants, only APOE4 allele reached significant values (Figure 2), with an effect size lower than that observed in other populations (p = 1.06E‐08; B = 1.42). Haplotype analysis of the APOE region revealed that two other variants (chr19:44906745:G:A and chr19:44918903:C:G), which have shown lower MAF in African than in Caucasian population, could change the APOE4 effect in our population (Figure 3).

**Conclusion:**

Our sample is composed by a heterogeneous genetic ancestry, different from the other Latin America populations. APOE4 allele presented a lower effect size to AD than some other populations. This result could be related to two other variants, associated with the African component, influencing the effect of APOE4 allele.